# Improving annotation propagation on molecular networks through random walks: introducing ChemWalker

**DOI:** 10.1093/bioinformatics/btad078

**Published:** 2023-03-02

**Authors:** Tiago Cabral Borelli, Gabriel Santos Arini, Luís G P Feitosa, Pieter C Dorrestein, Norberto Peporine Lopes, Ricardo R da Silva

**Affiliations:** NPPNS, Department of Molecular Biosciences, School of Pharmaceutical Sciences of Ribeirão Preto, University of São Paulo, Ribeirão Preto, SP 14040-903, Brazil; NPPNS, Department of Molecular Biosciences, School of Pharmaceutical Sciences of Ribeirão Preto, University of São Paulo, Ribeirão Preto, SP 14040-903, Brazil; NPPNS, Department of Molecular Biosciences, School of Pharmaceutical Sciences of Ribeirão Preto, University of São Paulo, Ribeirão Preto, SP 14040-903, Brazil; Collaborative Mass Spectrometry Innovation Center, Skaggs School of Pharmacy and Pharmaceutical Sciences, University of California, San Diego, La Jolla, CA 92093, USA; NPPNS, Department of Molecular Biosciences, School of Pharmaceutical Sciences of Ribeirão Preto, University of São Paulo, Ribeirão Preto, SP 14040-903, Brazil; NPPNS, Department of Molecular Biosciences, School of Pharmaceutical Sciences of Ribeirão Preto, University of São Paulo, Ribeirão Preto, SP 14040-903, Brazil

## Abstract

**Motivation:**

Annotation of the mass signals is still the biggest bottleneck for the untargeted mass spectrometry analysis of complex mixtures. Molecular networks are being increasingly adopted by the mass spectrometry community as a tool to annotate large-scale experiments. We have previously shown that the process of propagating annotations from spectral library matches on molecular networks can be automated using Network Annotation Propagation (NAP). One of the limitations of NAP is that the information for the spectral matches is only propagated locally, to the first neighbor of a spectral match. Here, we show that annotation propagation can be expanded to nodes not directly connected to spectral matches using random walks on graphs, introducing the ChemWalker python library.

**Results:**

Similarly to NAP, ChemWalker relies on combinatorial *in silico* fragmentation results, performed by MetFrag, searching biologically relevant databases. Departing from the combination of a spectral network and the structural similarity among candidate structures, we have used MetFusion Scoring function to create a weight function, producing a weighted graph. This graph was subsequently used by the random walk to calculate the probability of ‘walking’ through a set of candidates, departing from seed nodes (represented by spectral library matches). This approach allowed the information propagation to nodes not directly connected to the spectral library match. Compared with NAP, ChemWalker has a series of improvements, on running time, scalability and maintainability and is available as a standalone python package.

**Availability and implementation:**

ChemWalker is freely available at https://github.com/computational-chemical-biology/ChemWalker

**Contact:**

ridasilva@usp.br

**Supplementary information:**

Supplementary data are available at *Bioinformatics* online.

## 1 Introduction

The use of mass spectrometry-based metabolomics has greatly expanded over the last decade (Aksenov *et al.*, 2017). This expansion allowed a great improvement on *in silico* fragmentation spectral annotation, specially for methods based on machine learning (Schymanski *et al.*, 2017). The SIRUS software compiles multiple strategies for structural and chemical class annotation and has become one of the community’s key analysis tools, providing insight into the compound class and molecular formula when there are no spectral reference libraries that provide annotations (Hoffmann *et al.*, 2021). However, the structural annotations are performed at spectrum level, without taking in consideration the context information provided by the sample. We have previously shown that propagating the information by using multi-layered networks improves structural annotation (da Silva *et al.*, 2018). Using the propagation, combinatorial fragmentation (Ruttkies *et al.*, 2016) has shown great complementarity to SIRIUS (Di Ottavio *et al.*, 2020; Ernst *et al.*, 2019). The Network Annotation Propagation (NAP) had two main limitations: it only propagates information from direct neighbor nodes and it is available only as a webserver. Here, we present an improvement over NAP, using Random Walk on Graphs to allow information propagation in connected components of spectral networks (Grimmett, 2010).

## 2 Model description

The Random Walk on graphs selects an initial node $v_{0}$, and subsequently an adjacent node $v_{1}$ and moves to this neighbor (Grimmett, 2010). The walks can be determined by a probabilistic model with the following form:


(1) $$p_{ij}=P(X_{k+1}=j|X_{k}=i)=\{\begin{array}{ccc} \frac{w_{ij}}{w_{\left(i\right)}} & \text{if} & i,j\in E\\ 0 & & \text{otherwise} \end{array},$$

where $w_{\left(i\right)}=\sum_{j\in \Gamma (i)}w_{ij}$

, and *i* and *j* represent neighbor nodes in the graph. To maintain the MetFusion score setup (Gerlich and Neumann, 2013), averaging spectral and structural similarities, we applied the following formulation:


(2) $$w_{ij}=\alpha \frac{f_{i}+f_{j}}{2}+(1-\alpha )\text{sig}(m_{ij}t_{ij}),$$

where the indexes *i* and *j* represent two connected nodes, $f_{\text{index}}$ the MetFrag score for each node, the spectral similarity is represented by cosine score $m_{ij}$ between nodes *i* and *j*, multiplied by the chemical similarity $t_{ij}$ between MetFrag candidate structures associated to nodes *i* and *j*. The sig represents the sigmoid function.

### 2.1 Random walk algorithm

Following the description above, the algorithm to compute the candidate probabilities is given by

**Figure:**
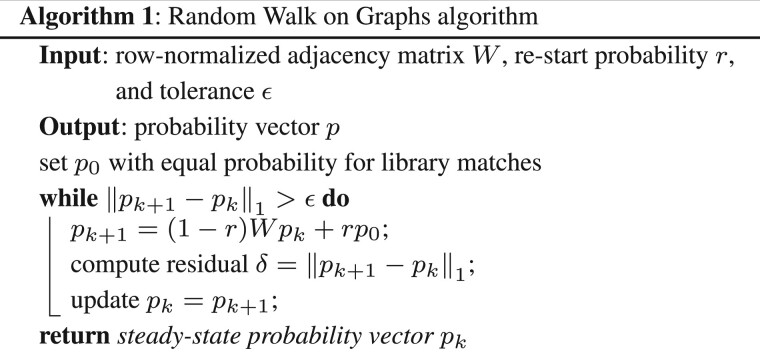


## 3 Implementation

The Random Walk model to propagate annotations, by re-ranking *in silico* annotations for spectra in molecular networks, was implemented in the python package ChemWalker. The API, or command line tool, allows the user to directly access Global Natural Product Social molecular networking - GNPS (Wang *et al.*, 2016) results and perform propagation inside connected components of the network (molecular families). To benchmark the model, a network composed of 555 spectra from NIST was used to compare 27 molecular fingerprints available at RDKit library (https://www.rdkit.org/). Comparing the classification of the 10 first candidates, it can be shown that ChemWalker cannot be statistically differentiated from NAP (Supplementary Material).

## 4 Conclusion

Taking in consideration that NAP’s benchmarking computed the improvement for annotation of all nodes, using information of all direct neighbors of these nodes, and that ChemWalker had at most 10% ‘seed nodes’ to propagate classification from (Supplementary Material), ChemWalker presents faster and more flexible propagation, allowing annotation improvements even when there is no direct neighbor with a spectral library match.

## Supplementary Material

btad078_Supplementary_Data

## Data Availability

All data used in the validation can be freely accessed through github repository: https://github.com/computational-chemical-biology/ChemWalker.
