# Correction to: Improving annotation propagation on molecular networks through random walks: introducing ChemWalker

**DOI:** 10.1093/bioinformatics/btad147

**Published:** 2023-03-24

This is a correction to: Tiago Cabral Borelli, Gabriel Santos Arini, Luís G P Feitosa, Pieter C Dorrestein, Norberto Peporine Lopes, Ricardo R da Silva, Improving annotation propagation on molecular networks through random walks: introducing ChemWalker, *Bioinformatics*, Volume 39, Issue 3, March 2023, btad078, https://doi.org/10.1093/bioinformatics/btad078

In the originally published online version of this manuscript, an inaccurate email was supplied for the corresponding author, Dr Da Silva: this should read: “ridasilva@usp.br”.

This error has been corrected in the article online.

