# Supplementary material for: Improving annotation propagation on molecular networks through random walks: introducing ChemWalker
Source: Bioinformatics. 2023 Mar 2;39(3):btad078. doi: 10.1093/bioinformatics/btad078 (PMC9991053; doi:10.1093/bioinformatics/btad078)
Supplement: btad078_Supplementary_Data [file btad078_supplementary_data.pdf]

Improving annotation propagation on molecular networks through random walks: Introducing ChemWalker

Tiago C. Borelli,[1] Gabriel Santos Arini,[1]

Luís G. P. Feitosa,[1] Pieter C. Dorrestein,[2]

Norberto P. Lopes,[1] and Ricardo R. da Silva[1*]

[1]NPPNS, Department of Molecular Biosciences

School of Pharmaceutical Sciences of Ribeirão Preto

University of São Paulo

[2]Collaborative Mass Spectrometry Innovation Center

Skaggs School of Pharmacy and Pharmaceutical Sciences

University of California

[*]To whom correspondence should be addressed.

Contact: ridasilva@usp.br

January 13, 2023

Abstract

Annotation of the mass signals is still the biggest bottleneck for the untargeted mass spectrometry analysis of complex mixtures. Molecular networks are being increasingly adopted by the mass spectrometry community as a tool to annotate large scale experiments. We have previously shown that the process of propagating annotations from spectral library matches on molecular networks can be automated using Network Annotation Propagation (NAP). One of the limitations of NAP is that the information for the spectral matches is only propagated locally, to the first neighbor of a spectral match. Here we show that annotation propagation can be expanded to nodes not directly con-

nected to spectral matches using random walks on graphs, introducing the ChemWalker python library. Similarly to NAP, ChemWalker relies on combinatorial in silico fragmentation results, performed by MetFrag, searching biologically relevant databases. Departing from the combination of a spectral network and the structural similarity among candidate structures, we have used MetFusion Scoring function to create a weight function, producing a weighted graph. This graph was subsequently used by the random walk to calculate the probability of 'walking' through a set of candidates, departing from seed nodes (represented by spectral library matches). This approach allowed the information propagation to nodes not directly connected to the spectral library match. Compared to NAP, ChemWalker has a series of improvements, on running time, scalability and maintainability and is available as a stand alone python package. ChemWalker is freely available at https://github.com/computational-chemical-biology/ChemWalker.

1 Model description

The MetFusion [1] model uses spectral similarity to inform *in silico* fragmentation prediction. The approach re-calculates the MetFrag score using the following formulation

$$S_c = \alpha f_c + (1 - \alpha) \sum_{j=1}^{M} sig(m_j t_{cj}) \quad (1)$$

where the index c represents each MetFrag candidate, f_c the MetFrag [2] score and the spectral similarity is represented by the spectral library match score m_j for all j neighbor nodes, multiplied by the chemical similarity t_{cj} between MetFrag candidates c associated to each neighbor node j. The sig represents the sigmoid function.

The Network Annotation Propagation (NAP) model uses the MetFusion concept to propagate annotations from spectral library matches through the network topology [3]. The Figure 1 illustrates the propagation concept.

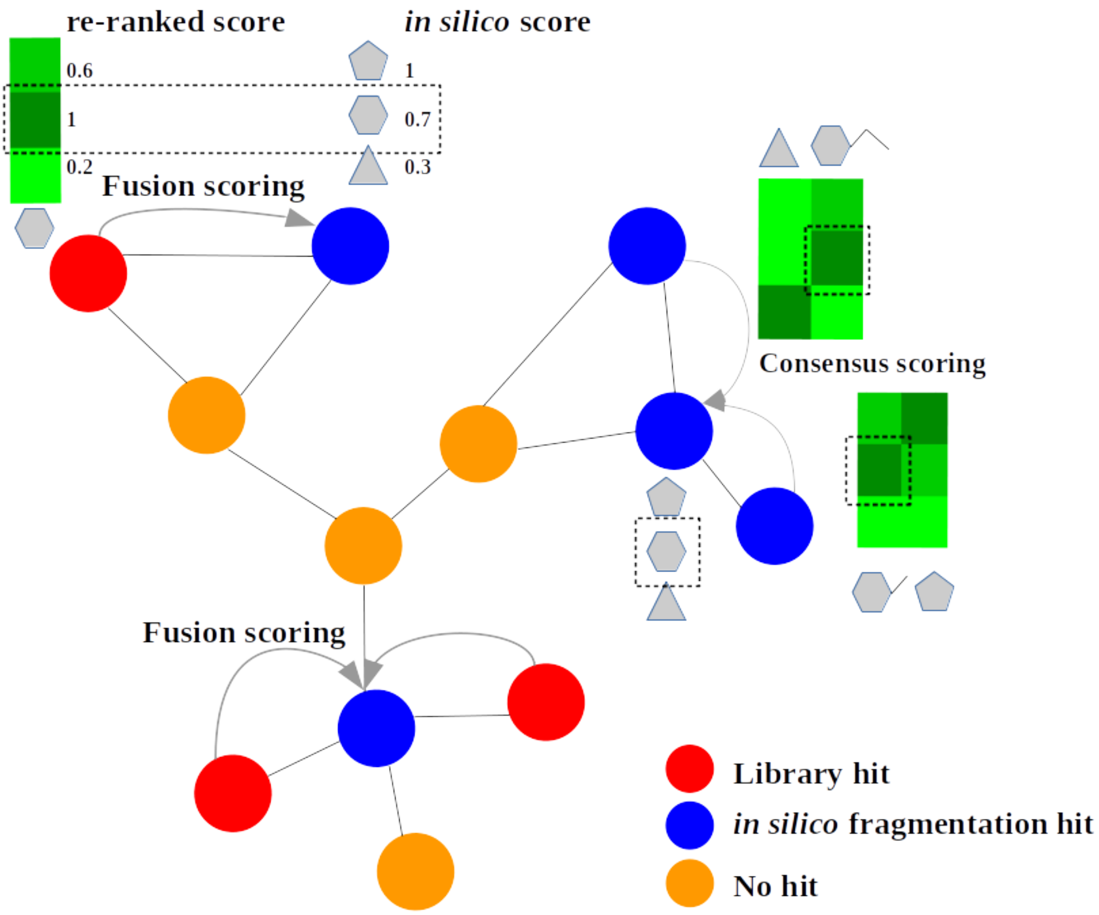

Figure 1: Network Annotation Propagation. The *Fusion* scoring uses the reference from spectral similarity to evaluate structural similarity to candidate structures and re-rank the candidates. The *Consensus* scoring uses the similarity among candidates to retrieve sets of similar candidates.

1.1 Structural similarity graph

From NAP's model one can derive a graph of structural similarity, following the spectral network topology. The Figure 2 illustrates the replacement of the spectra, by its *in silico* fragmentation candidates and the generation of a structural similarity graph, where the edges are weighted by a similarity score, discussed below.

The *Graph* $G = (N, E)$ can be represented by a set of $|N| = n$ nodes, representing the candidate structures, and $|E| = e$ edges representing the structural similarity between two given candidates.

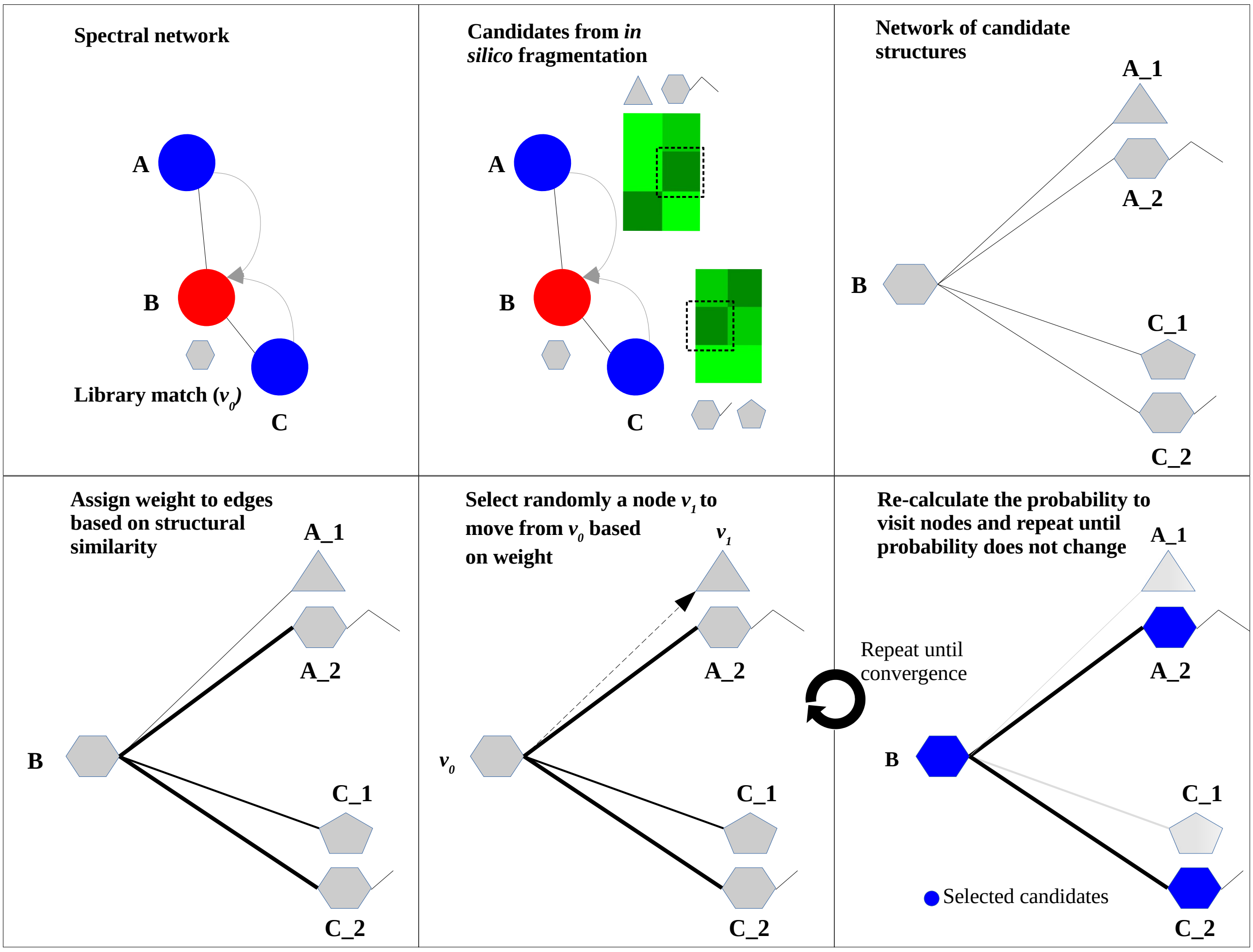

Figure 2: Schematic representation of ChemWalker's algorithm. The similarity between candidates of the neighbor nodes are used to create a weighted graph. A sequence of nodes is selected (e.g. from v_0 to v_1) and the probability to move to a given node is calculated based on weights. The process is repeated until there is no change in probability (convergence).

1.2 Random Walk model

Under the assumption that the spectral similarity observed among the spectra infers the structural similarity of the underlying compound structures, the Random Walk on graphs selects an initial node v_0, and subsequently an adjacent node v_1 and moves to this neighbor [4]. The walks can be determined by a probabilistic model with of the following form

$$p_{ij} = P(X_{k+1} = j \mid X_k = i) = \begin{cases} \frac{w_{ij}}{w_{(i)}} & if \quad i, j \in E \\ 0 & \quad otherwise \end{cases} \tag{2}$$

where $w_{(i)} = \sum_{j \in \Gamma(i)} w_{ij}$, and i and j represent neighbor nodes in the graph. To maintain the MetFusion score setup, averaging spectral and structural similarities, we applied the following formulation

$$w_{ij} = \alpha \frac{f_i + f_j}{2} + (1 - \alpha) sig(m_{ij} t_{ij}) \tag{3}$$

where the indexes i and j represent two connected nodes, f_{index} the MetFrag score for each node, the spectral similarity is represented by cosine score m_{ij} between nodes i and j, multiplied by the chemical similarity t_{ij} between MetFrag candidate structures associated to nodes i and j. The sig represents the sigmoid function. The Markov property holds, where conditional on the present, the future is independent of the past, therefore:

$$P(X_{k+1} = j \mid X_k = i, X_{k-1} = i_{k-1}, \ldots, X_0 = i_0) = P(X_{k+1} = j \mid X_k = i) = p_{ij} \tag{4}$$

The probability of choosing a given *i-th* candidate structure at a step k of the Random Walk is given by the probability vector p_k [5], which at any given step can be obtained by

$$p_{k+1} = (1 - r) W p_k + r p_0 \tag{5}$$

where W is the column-normalized adjacency matrix of the graph, p_0 was constructed such that equal probabilities were assigned to the nodes representing 'seed' spectral library matches and r is the restart probability, which restricts on how far we want the random walker to get away from the start 'seed' node(s). The candidate compounds are ranked according to the values in the steady-state probability vector p_∞. This state is obtained at query time by performing the iteration until the change between p_k and p_{k+1} (measured by the L1 norm) falls below 10^{-6}.

1.3 Random Walk algorithm

Following the description above the algorithm to compute the candidate probabilities is given by:

Algorithm 1: Random Walk on Graphs algorithm

Input: row-normalized adjacency matrix W, re-start probability r, and tolerance ϵ

Output: probability vector p

set p_0 with equal probability for library matches

while $\|p_{k+1} - p_k\|_1 > \epsilon$ **do**

  $p_{k+1} = (1 - r)Wp_k + rp_0$;

  compute residual $\delta = \|p_{k+1} - p_k\|_1$;

  update $p_k = p_{k+1}$;

return *steady-state probability vector* p_k

2 Benchmarking ChemWalker

In order to benchmark NAP, 5,467 spectra from NIST library were used to compare the propagation scores to MetFrag used on each spectra individually. To extend the benchmark, a subset of 555 spectra, keeping the proportionality of the NAP's result, as shown in the comparison between the subset and the complete dataset (Figure 3), was selected [3], to compare the previous results with ChemWalker.

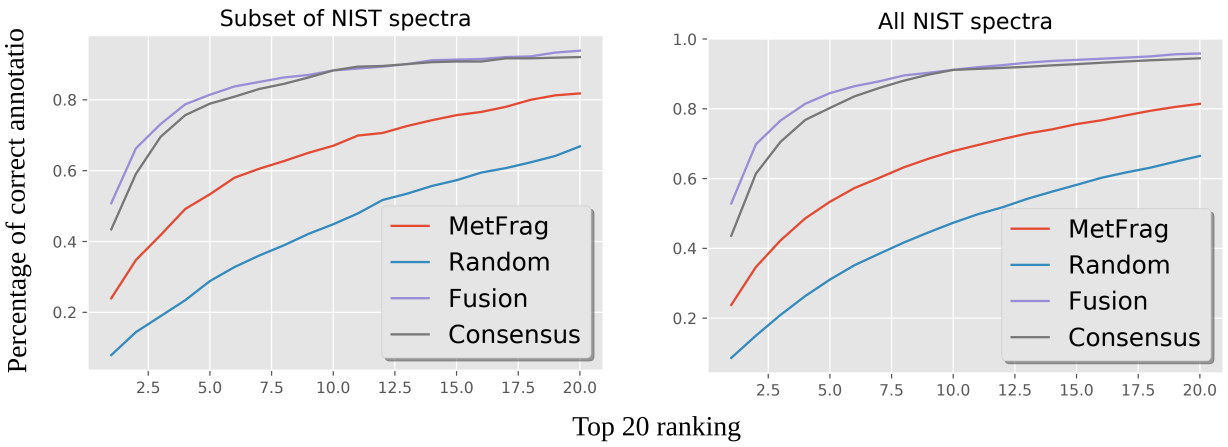

Figure 3: Spectra sub-set from NAP's validation used to ChemWalker comparison.

First, the molecular fingerprint used for propagation was compared. The RDKit (`https://www.rdkit.org/`) library was used, and 27 fingerprints were compared. As ChemWalker was designed to work with all spectral library matches as seed nodes, we established 10% of nodes of a given connected component to be the number of seeds in the benchmark dataset (one node for connected components with less than 10 nodes). The fingerprint comparison is shown on Figure 4.

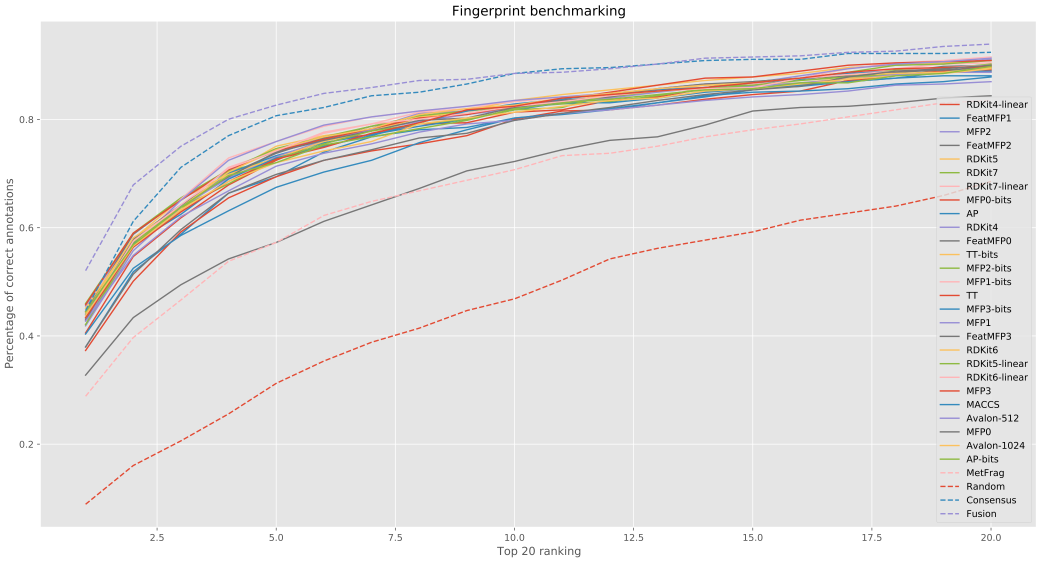

Figure 4: Fingerprint comparison for ChemWalker, using RDKit library. The curves labeled *Consensus* and *Fusion* were obtained by by NAP, all other curves from RDKit fingerprints were obtained by ChemWalker.

Despite the improvement over Random and MetFrag, and a higher number of correct candidates ranked in the first three position, comparing *Consensus* scoring with the fingerprints *MFP2*, *TT-bits*, *MFP2-bits* and *TT*, the visual inspection shows an overall superiority for *Consensus* and *Fusion* scoring. This was expected, since all neighbors of all nodes act as additional information for these scores, while, for ChemWalker, there are connected components ranging from 346 to 2 nodes, and the only information available comes from seed nodes. Figure 5 shows a connected component of 15 nodes, where 11 were improved over MetFrag. The two *seed* nodes where randomly placed, and we can see an example of improvement even at 3 nodes distance (distance from lower blue border *seed*, node 57 to top green border improved classification, node 159). The benchmark analysis can be found at *benchmark.ipynb* on ChemWaker's git repository.

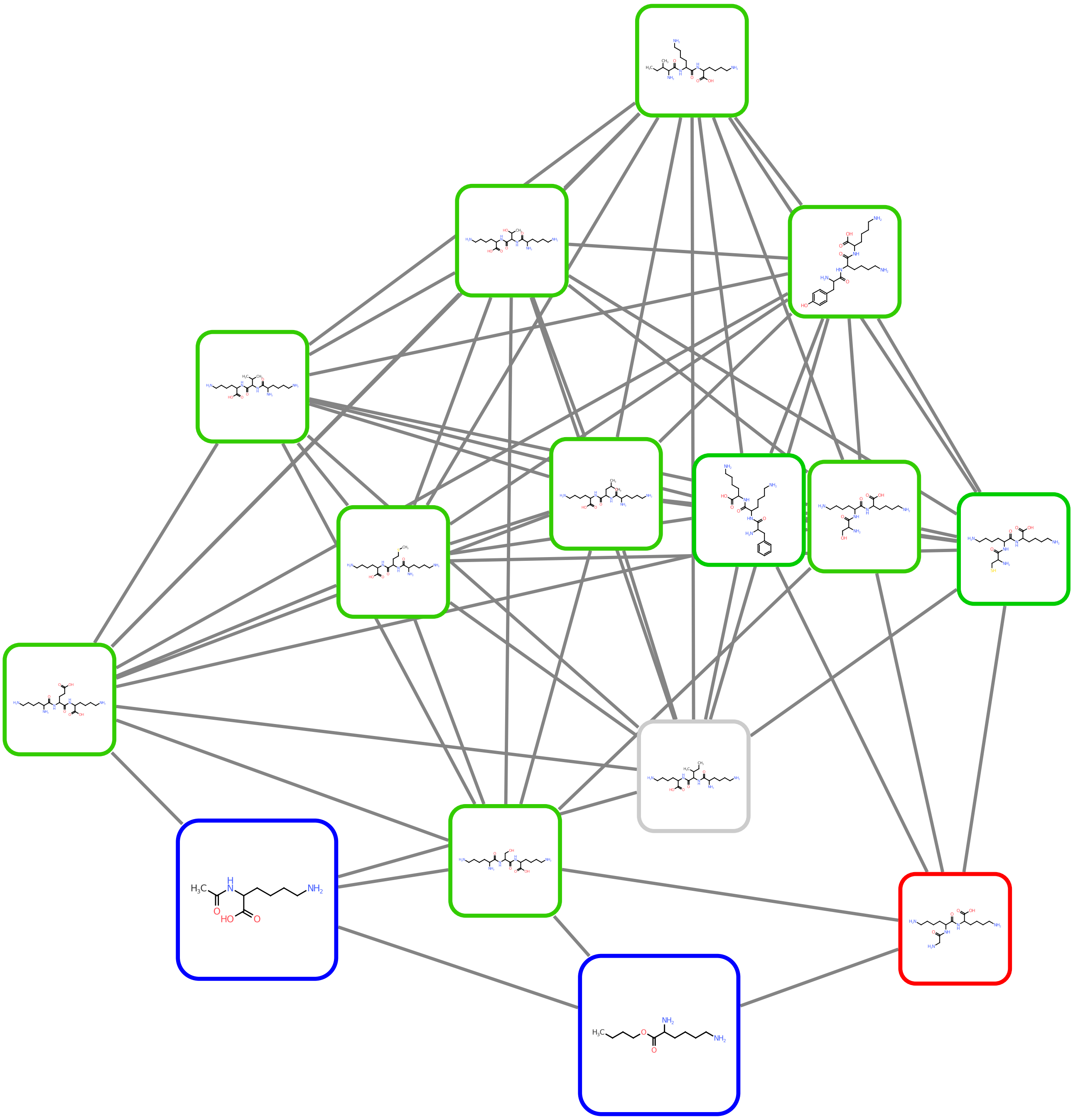

Figure 5: Connected component analysed by ChemWalker. Larger nodes represent *seed* spectral library matches, from where random walks re-start. Green borders represent improvement over MetFrag, red nodes represent loss over MetFrag and grey no ranking alteration.

Carefully inspecting some of the best performing fingerprints, we observe in Figure 6, that the difference between the performance improvement of ChemWalker over MetFrag seems compatible to *Consensus* and *Fusion* scoring.

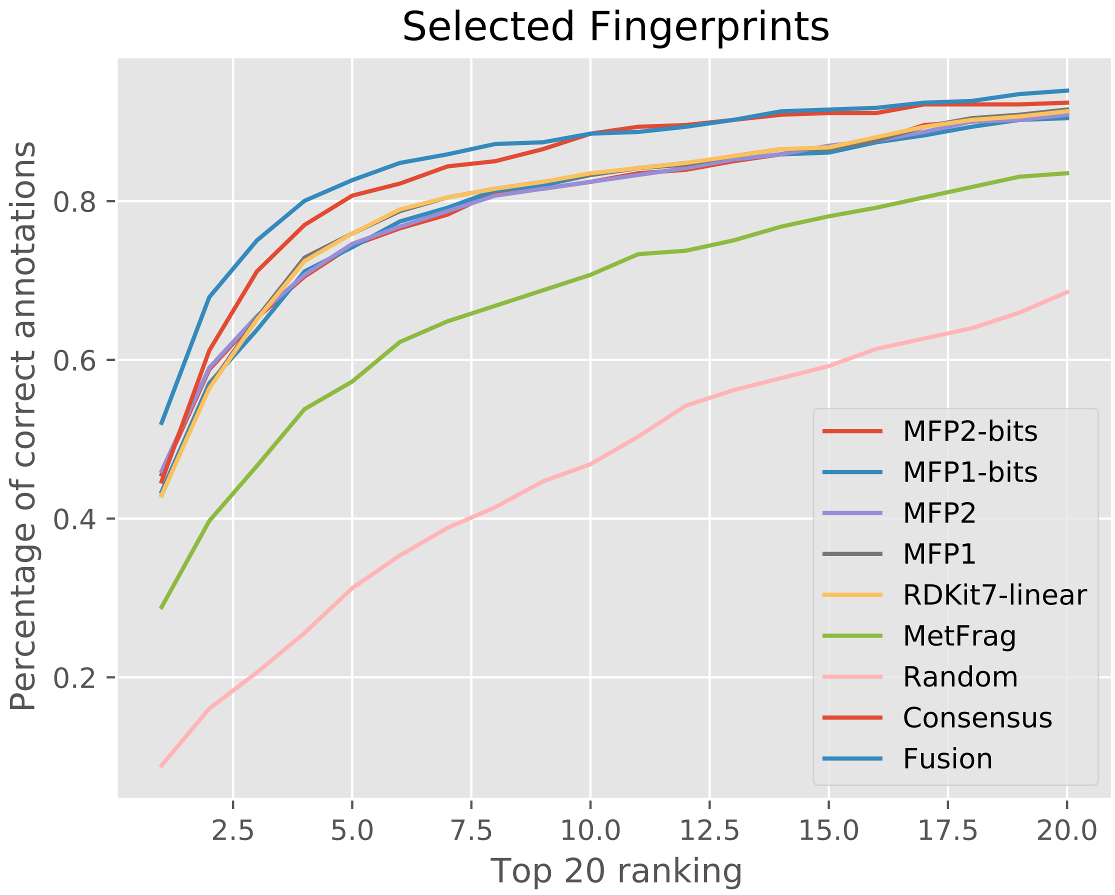

Figure 6: Fingerprint comparison for ChemWalker, using selected RDKit library fingerprints. The curves labeled *Consensus* and *Fusion* were obtained by by NAP, all other curves from RDKit fingerprints were obtained by ChemWalker.

Using Chi-square to test the goodness of fit for the number of candidates classified in the 20 first positions, between ChemWalker with different fingerprints and NAP, we can see that some of the best performing fingerprints cannot be statistically differentiated from *Consensus* and *Fusion* scores (Table 1).

Table 1: Comparison between ChemWalker, with selected fingerprints, to NAP's scores.

Fingerprint	Score	chi-square statistic	p-value
RDKit7-linear	Random	164.640	$3.168e^{-25}$
	MetFrag	33.413	0.021
	Consensus	19.538	0.423
	Fusion	19.881	0.402
MFP2	Random	168.793	$4.895e^{-26}$
	MetFrag	43.221	0.001
	Consensus	27.192	0.100
	Fusion	20.450	0.368
MFP2-bits	Random	164.238	$3.794e^{-25}$
	MetFrag	40.116	0.003
	Consensus	22.338	0.268
	Fusion	20.091	0.389
MFP1-bits	Random	165.005	$2.688e^{-25}$
	MetFrag	31.823	0.033
	Consensus	15.821	0.669
	Fusion	22.685	0.251
MFP1	Random	164.081	$4.071e^{-25}$
	MetFrag	34.725	0.015
	Consensus	13.973	0.785
	Fusion	19.364	0.434

References

[1] Michael Gerlich and Steffen Neumann. "MetFusion: integration of compound identification strategies". In: *Journal of Mass Spectrometry* 48.3 (2013), pp. 291–298. DOI: `https://doi.org/10.1002/jms.3123`. eprint: `https://analyticalsciencejournals.onlinelibrary.wiley.com/doi/pdf/10.1002/jms.3123`. URL: `https://analyticalsciencejournals.onlinelibrary.wiley.com/doi/abs/10.1002/jms.3123`.

[2] Christoph Ruttkies et al. "MetFrag relaunched: incorporating strategies beyond in silico fragmentation". en. In: *Journal of Cheminformatics* 8.1 (Jan. 2016), p. 3. ISSN: 1758-2946. DOI: `10.1186/s13321-016-0115-9`. URL: `http://www.jcheminf.com/content/8/1/3/abstract`.

[3] Ricardo R. da Silva et al. "Propagating annotations of molecular networks using in silico fragmentation". In: *PLOS Computational Biology* 14.4 (Apr. 2018). Ed. by Avner Schlessinger, e1006089. ISSN: 1553-7358. DOI: `10.1371/journal.pcbi.1006089`. URL: `http://dx.plos.org/10.1371/journal.pcbi.1006089`.

[4] Geoffrey Grimmett. *Probability on Graphs: Random Processes on Graphs and Lattices*. Institute of Mathematical Statistics Textbooks. Cambridge University Press, 2010. DOI: 10.1017/CBO9780511762550.

[5] Sebastian Kohler et al. "Walking the Interactome for Prioritization of Candidate Disease Genes". In: *The American Journal of Human Genetics* 82.4 (Apr. 2008), pp. 949–958. ISSN: 0002-9297. DOI: 10.1016/J.AJHG.2008.02.013. URL: https://www.sciencedirect.com/science/article/pii/S0002929708001729.
